# Mind Your Data: Privacy and Legal Matters in eHealth

**DOI:** 10.2196/17456

**Published:** 2021-03-17

**Authors:** Catharina M L Zegers, Annemieke Witteveen, Mieke H J Schulte, Julia F Henrich, Anouk Vermeij, Brigit Klever, Andre Dekker

**Affiliations:** 1 Digital Society Health & Well-being The Hague Netherlands; 2 Institute of Data Science Maastricht University Maastricht Netherlands; 3 Department of Radiation Oncology (MAASTRO) GROW - School for Oncology and Development Biology Maastricht University Medical Centre Maastricht Netherlands; 4 Department of Biomedical Signals and Systems TechMed Centre University of Twente Enschede Netherlands; 5 Department of Clinical, Neuro and Developmental Psychology Vrije Universiteit Amsterdam Netherlands; 6 Unit of Health, Medical and Neuropsychology Institute of Psychology Leiden University Leiden Netherlands; 7 Department of Cognitive Neuropsychology Tilburg University Tilburg Netherlands; 8 University Medical Center University of Groningen Groningen Netherlands

**Keywords:** data, privacy, eHealth

## Abstract

The health care sector can benefit considerably from developments in digital technology. Consequently, eHealth applications are rapidly increasing in number and sophistication. For successful development and implementation of eHealth, it is paramount to guarantee the privacy and safety of patients and their collected data. At the same time, anonymized data that are collected through eHealth could be used in the development of innovative and personalized diagnostic, prognostic, and treatment tools. To address the needs of researchers, health care providers, and eHealth developers for more information and practical tools to handle privacy and legal matters in eHealth, the Dutch national Digital Society Research Programme organized the “Mind Your Data: Privacy and Legal Matters in eHealth” conference. In this paper, we share the key take home messages from the conference based on the following five tradeoffs: (1) privacy versus independence, (2) informed consent versus convenience, (3) clinical research versus clinical routine data, (4) responsibility and standardization, and (5) privacy versus solidarity.

## The Digital Society Program

The Association of Universities in the Netherlands (VSNU) has brought together scientists from all 14 universities in the Netherlands to address the pressing questions raised by the emergence of a digital society. Exploring the responsible use of innovative digital technologies in the health care sector has high societal priority because of the potential to significantly improve health care and reduce health care costs. The Health & Well-Being program line of the VNSU Digital Society Research Programme aims to develop, evaluate, and implement integrated and personalized digital health care solutions, while addressing the societal challenges raised by the digitalization of health care.

Decelerating factors in the development and implementation of eHealth are a lack of knowledge, information, and practical tools with respect to handling privacy and legal matters. To discuss these factors, the Digital Society Health & Well-Being team organized a conference on September 26, 2019 titled “Mind Your Data: Privacy and Legal Matters in eHealth” with the aim to learn from each other’s approaches to tackle privacy and legal matters in the development of eHealth.

The conference hosted five speakers who were selected based on their unique backgrounds (law, eHealth, data science, philosophy, and mobile health [mHealth]), vision, and expertise on privacy and legal issues in eHealth. Marie-José Bonthuis is an external privacy lawyer who is connected to the Medical Biobank Lifelines and to the University Medical Center Groningen. Furthermore, she is an expert in the Health Research Infrastructure initiative (Health-RI) service desk for ethical, legal, and societal questions related to personalized medicine and next-generation sequencing. Dr. Bonthuis presented a talk titled “Overview of data protection principles in research: bringing practice and legislation together.” Niels Chavannes is a professor of Public Health and Primary Care at Leiden University Medical Centre, a general practitioner, and the founder of the National eHealth living lab (NeLL). Professor Chavannes presented a talk titled “Clinical implementation of successful eHealth initiatives: ethical and legal issues.” Andre Dekker is the professor of Clinical Data Science at Maastricht University, Maastricht University Medical Center+, and MAASTRO Clinic. Professor Dekker presented a talk titled “The personal health train: privacy preserving learning from health data.” Peter Paul Verbeek is the professor of Philosophy of Technology and scientific codirector of DesignLab of the University of Twente. In addition, he is an honorary professor of Techno-Anthropology at Aalborg University and chair of the UNESCO World Commission for the Ethics of Science and Technology. Professor Verbeek shared his perspective in a talk titled “Privacy and beyond: inclusive digitalisation and the dynamics of privacy.” Finally, Edward Watkins is the professor of Experimental and Applied Clinical Psychology at the University of Exeter. Professor Watkins presented a talk titled “ECoWeB – mental health app for young people data and governance issues.”

More than 100 participants from a wide range of organizations (universities, medical centers, knowledge institutes, private parties, citizens, and government) attended the conference. Three independent authors noted down specific points that were expressed during the presentations, panel discussion, and eHealth forum. These notes were compared, sorted in categories, and juxtaposed in a way that the ethical challenges clearly emerged. Solutions provided by speakers were described; otherwise, clarification was provided by the authors of the paper. This resulted in our summary of the most prominent ethical-, technical-, and research-related issues in eHealth and their potential solutions.

## Privacy Versus Independence

There is no straightforward answer for the best way to address privacy issues in eHealth. For each eHealth application, there should be a balance between individual privacy and potential individual or societal benefit. Data protection is all about contextual integrity; that is, using data responsibly within a specific context. Take for example the development of an mHealth approach to assess and enhance emotional competence for well-being in the young (ECoWeB project) [1]. In this project, young people expressed their reluctance to share passive sensor data and preferred to be in control of the data they would like to release. In this situation, an active approach to gather data (eg, by questionnaires) is most suitable. By contrast, the use of cameras, a more privacy-intrusive method, is more likely to be accepted in smart homes for people with dementia, since the benefit for the user is larger, as people could have the opportunity to stay at home for a longer time [2]. This is in agreement with Wilkowska et al [3], who showed that female patients and healthy adults insist on higher security and privacy standards, whereas people with ailing health consider privacy of lower importance. End users seem to be willing to tradeoff part of their privacy for the benefit of, for example, independence.

To gain insight into this tradeoff, user preferences, and needs, it is essential to include the end user in the design of eHealth at an early stage of development. This should provide an understanding as to what extent the user is willing to share data and for what purpose. During the conference, this was exemplified by the ECoWeB project [1] in which co-design with young people was critical. The data collection and recruitment are fully online processes, and the use of data is transparent and clear to the users. Previous research has shown that the uptake of eHealth and mHealth is only successful when they are built to fulfill a certain need of either patients or health care providers [4]. The early involvement of clinicians and patients will encourage adoption and maximize the positive impact of an intervention [5]. An example of a dedicated approach to develop and evaluate eHealth is the Centre for eHealth and Wellbeing Research roadmap developed by van Gemert et al [6], which focuses on user participation and process evaluation.

## Informed Consent Versus Convenience

eHealth research generally includes an informed consent procedure for use and accessibility of data. This can potentially be done by digital authentication, including, for example, parental consent and age verification. However, during the conference’s panel discussion, the issue about how elaborate digital informed consent should be arose. The panelists concluded that there should be a balance between simple, convenient, and easy to understand versus fully complete. This tradeoff is similar to a paper-based consent procedure. Nevertheless, there seems to be a striking difference between the requirements for informed consent of eHealth in comparison to commercial applications. An editorial published in *Nature* [7] also addressed this issue, stating that the consent for commercial mobile applications is often not more than a box to tick, with terms and conditions that are hardly ever read by the users.

In addition to informed consent, it is highly important to address the expectations of the eHealth app. This includes information on the procedure for incidental findings, such as whether or not the user wants to be actively informed or what can be expected with regard to automated messaging/triggering the health care provider for actions in the case of a monitoring app. Providing this additional information might limit possible overexpectations of users of the app.

## Clinical Research Versus Clinical Routine Data

Data obtained after informed consent are only available from a small population of people that are registered for clinical research or use a specific eHealth app. By contrast, general registries collect data from a large number of patients, but the information is limited to demographics and a small selection of clinical variables. Another data source is clinical routine data, which contains the largest amount of clinically relevant information. One could think of a “patients like me” approach, where we can learn from existing data worldwide to find a similar patient. Unfortunately, clinical routine data are very hard to collect centrally because they are stored in individual local databases. One of the potential solutions is the use of distributed learning. The Personal Health Train is an example of this, where the data remain at the source (eg, the hospital) in Findable, Accessible, Interoperable, Reusable (FAIR) data stations, and the analysis method (eg, the algorithm) is transferred to the data. This method has been successfully implemented such as for predicting the 2-year survival of lung cancer patients using clinical data of 20,000 patients [8,9].

To allow for the secondary use of clinical personal data, data should be made nonidentifiable or anonymous [10], however, it is unclear when data are truly anonymous. Complete anonymity cannot be guaranteed if combinations of demographic data are given, and thus the question as to when data are truly anonymous remains. Many datasets that appeared to be anonymous have been released and individuals were reidentified [11]. Reidentification is generally performed by media or researchers, with the aim to show that shared data are unsafe, to publish new algorithms, and show weaknesses in the databases. An example was the reidentification of an individual from an adverse events database of Health Canada, and the media were able to reidentify a deceased woman based on a match of age, location, and date of death [12]. Algorithms such as k-anonymity can be used to describe the level of anonymity of datasets that plays a role in the definition of (non)identifiable personal data [13]. In addition, to determine the likelihood of an individual to be correctly reidentified, Rocher et al [11] proposed and validated a statistical model that was able to reidentify individuals even if the dataset was heavily incomplete. As such, if a dataset has been completely anonymized, it would be impossible to find the data of an individual who would like to withdraw consent to use their data. This is problematic if participants have been informed that they can withdraw their consent at any time [7]. An additional problem occurs in research scenarios that require data to be linked across different entities (eg, linking medical data from a hospital to socioeconomic data from Statistics Netherlands). These scenarios demand separate solutions that address privacy while still enabling subject-level linking based on common information [14].

## Responsibility and Standardization

There are no straightforward answers to the questions of who is responsible for digital health apps, and how to guarantee maximal privacy and compliance with legislation. Together with multiple partners, the VSNU developed a Code of Conduct for research integrity in the Netherlands in 2018. The responsibilities have been defined at multiple levels, from the individual researcher to the boards of research institutions and the institutions as a whole [15]. Researchers can also use the General Data Protection Regulation (GDPR) carwash [16], a prototype of a practical flowchart that aims to guide researchers through the necessary steps to work with personal data in a GDPR-compliant manner.

Moreover, it is hard for a user to determine which app is qualitatively good. The availability of health apps is increasing rapidly. Pereira-Azevedo and Venderbos [4] estimated that over 300,000 medical apps were available in 2018. Medical App Checker developed by the Royal Dutch Medical Association was established as an initiative to evaluate mobile medical apps [17]. Medical App Checker consists of several checklists, including one for evaluating the protection and security of personal data. Additionally, an international norm (ISO standard) is in development for health and wellness apps. This standard could be used to certify apps that meet the norms for safety, reliability, and user-friendliness based on existing quality requirements and legislation. The latter is in flux as the new EU Medical Device Regulation, which takes effect on May 2021, will set more stringent demands on medical applications.

Finally, when moving from research toward the clinical implementation of eHealth, Dutch professional communities (medical specialists, medical physics, and clinical informatics) have expressed in their vision statements that they will take their responsibilities in the stimulation of the development and use of eHealth, and to assure its quality and safety.

## Privacy and Solidarity

Technological innovations change our society rapidly and the interaction of humans with these digital innovations may also influence our perception of societal values such as privacy. The complex interactions of how innovations influence the ethical frameworks with which they are valued can be exemplified with a Google Glass study. In this study, a technological mediation approach was used to focus on the dynamics of the interaction between technologies and human values. Online discussions about Google Glass technology were investigated to evaluate how people articulate new meanings of the value of privacy [18].

Additionally, there are cultural differences in the way we value privacy, especially on a global scale. To account for this dynamism of values, value-sensitive and responsible design approaches should be adopted. There is also a movement toward solidarity and data donorship. Toward this end, a culturally sensitive balance should be sought between sharing (“give data and save lives“) and protection (eg, potential threat of commercial exploitation) of data.

## Conclusion

The information presented and discussed at the conference highlighted the many tradeoffs in eHealth with regard to privacy and legal questions. To prevent potential decelerating factors in the development and implementation of eHealth, we need to be aware of these tradeoffs between (i) privacy and independence, (ii) informed consent and convenience, (iii) clinical research and clinical routine data, (iv) responsibility and standardization, and (v) privacy and solidarity. Furthermore, we need to make use of the available knowledge and tools on a national and international level, think carefully about the design of the application, and include end users at an early stage of development to reach the full potential of the eHealth technology. Clearly, there are risks associated with developments in eHealth, but rather than avoiding risks and stalling innovation, we should attempt to minimize risks while providing the greatest possible benefits to society.

## Abbreviations

ECoWeB: emotional competence for well-being in the young
FAIR: Findable, Accessible, Interoperable, Reusable
GDPR: General Data Protection Regulation
Health-RI: Health Research Infrastructure Initiative
mHealth: mobile health
VSNU: Association of Universities in the Netherlands
